# Industrial effluent-derived *Acinetobacter* sp. PbS1A2 as a bioremediator against lead toxicity in *Labeo rohita*

**DOI:** 10.1128/aem.00527-26

**Published:** 2026-08-21

**Authors:** Saima Zafar, Asma Ashraf, Sehrish Fatima, Farkhanda Asad, Kafeel Ahmad, Yusuf Tutar, Mudassir Hassan

**Affiliations:** 1 Department of Zoology, Government College University Faisalabad 72594, Faisalabad, Punjab, Pakistan; 2 Department of Botany, Baba Guru Nanak University 686577, Nankana Sahib, Punjab, Pakistan; 3 Division of Biochemistry, Faculty of Medicine, Recep Tayyip Erdogan University 485660, Rize, Turkey; 4 Department of Zoology, Baba Guru Nanak University 686577, Nankana Sahib, Punjab, Pakistan; University of Milano-Bicocca, Milan, Italy

**Keywords:** isolation, bioremediation, growth parameters, minimum inhibitory concentration, histopathology

## Abstract

Lead pollution is recognized as a major global environmental problem that poses a serious threat to ecological integrity and environmental sustainability. The current study shows the bioremedial potential of Pb-resistant *Acinetobacter* sp. PbS1A2 in *Labeo rohita*. The fish were divided into three experimental treatments, namely, Control (T_0_), Pb only (T_1_), and Pb + *Acinetobacter* sp. (T_2_). The treatment T_2_ was particularly exposed to a concentration of 1.5 × 10^8^ CFU/mL of *Acinetobacter* sp. along with Pb (1 mg L^−1^) for a period of 30 days. The results revealed that *Acinetobacter* sp. PbS1A2 could tolerate Pb concentrations up to 30 mM, along with resistance to other heavy metal ions. Pb exposure significantly increased the activity of all antioxidant enzymes, with glutathione transferase exhibiting the greatest increase (795%), followed by peroxidase (229%), superoxide dismutase (195%), catalase (104%), and ascorbate peroxidase (28%). Furthermore, Fourier transform infrared spectroscopy was employed to identify the functional groups involved in Pb biosorption, while intracellular accumulation was confirmed via energy dispersive X-ray and scanning electron microscopy analyses. At the end of the 1-month trial, fish in the T_2_ group treated with *Acinetobacter* sp. exhibited a significantly higher survival rate, improved growth performance, better body composition, and increased amylase and protease activities than those in the T_1_ group. Furthermore, the T_2_ treatment mitigated the negative impacts of Pb on hematological and biochemical parameters and restored histological damage in the fish organs. The current study suggests that *Acinetobacter* sp., due to its heavy metal tolerance, could effectively ameliorate Pb bioavailability in aquatic habitats and enhance environmental health.

**IMPORTANCE:**

The continuously rising Pb discharge in aquatic ecosystems, particularly in the industrial regions of South Asia, jeopardizes ecological integrity and the health of economically crucial fish such as *Labeo rohita*, consequently affecting food security. Conventional remediation techniques are often expensive, energy-intensive, and pose ecological risks. This study addresses a significant gap by presenting a thorough bioremediation process encompassing the isolation and characterization of a newly identified multi-metal-resistant *Acinetobacter* sp. (PbS1A2) from industrial wastewater, which facilitates *in vivo* recovery in *Labeo rohita* contaminated with Pb. Isolated from a contaminated site, *Acinetobacter* sp. have evolved natural metal-resistant mechanisms under environmental stress, resulting in better remediation than lab-engineered options. This is distinguished from studies that solely address the *in vitro* efficiencies of Pb removal. The research offers extensive biological evidence indicating that Pb-induced damage can be effectively mitigated. The results concerning growth performance, hematological assessments, digestive enzyme activities, tissue accumulation, and histopathological evaluations suggest an economical, expandable, and scientifically validated approach to manage Pb pollution in aquatic ecosystems.

## INTRODUCTION

In recent decades, urban Pb concentrations have increased globally, worsening its detrimental effects on ecosystems and the human population (1). Pb concentrations in urban soils in 174 cities worldwide (2010–2022) peaked at 5,780.00 mg/kg, much above permissible levels, highlighting an alarming global pollution problem (2). Pb’s pervasive presence in the environment stems from its diverse sources, including industrial activities like smelting, automobile and battery production, as well as natural processes like volcanic eruptions and weathering (3). Numerous health problems are associated with Pb exposure. It leads to cardiovascular, neurological, skeletal, reproductive, hematological, and renal disorders in adults and neurological and developmental issues in children (4). About 65% of human Pb exposure occurs from contaminated meals and fish (5). The contamination of aquatic ecosystems causes Pb accumulation in fish, which is naturally stimulated by variables like temperature, salinity, concentration, species, age, size, sex, nutrition, and metabolism (6). This accumulation causes behavioral changes, slows growth, and alters physicochemical factors like enzymatic activity, histopathology, hematobiochemical parameters, and oxidative stress balance (7). These biological impacts of Pb, along with its widespread environmental occurrence, reflect the broader vulnerability of aquatic ecosystems to multiple pollutants (3).

As a result, a variety of pollutants, notably heavy metals, pesticides, industrial effluents, and toxins from shipping and port operations, are posing a rising threat to aquatic ecosystems. These pollutants seriously impair water quality and upset the biological balance of aquatic environments. Because of their persistence, non-biodegradability, and propensity to bioaccumulate in aquatic organisms, heavy metals like arsenic (As), cadmium (Cd), chromium (Cr), lead (Pb), and copper (Cu) are among the most dangerous pollutants. They cause oxidative stress, hematological and biochemical disorders, tissue damage, and compromised physiological functions (8–11). Similarly, pesticide residues entering aquatic habitats through agricultural runoff also have a negative impact on fish health by affecting microbiological, biochemical, and metabolic processes, lowering organismal fitness and ecosystem stability (12). Environmental monitoring further demonstrated the accumulation of hazardous metals in edible freshwater fish, underlining both ecological hazards and possible threats to human health through the aquatic food chain (13). Furthermore, rising maritime transportation and port operations add to aquatic pollution by releasing oils, chemicals, ballast water, and other contaminants, leading to habitat deterioration and biodiversity loss (14). These pollutants together represent significant environmental threats to aquatic environments and aquatic species, underlining the necessity for continual monitoring and sustainable management of aquatic ecosystems (15).

In response to these escalating environmental concerns, various remediation strategies have been developed to remove Pb from contaminated aquatic systems. Physicochemical techniques like chemical precipitation, ion exchange, adsorption, and membrane processes for Pb pollution removal have gained a lot of interest recently (16). However, these techniques have serious disadvantages, such as low removal efficiency, high-energy costs, and sludge production that call for extra resources (17). As a result, research for more sustainable and effective technology is needed (18). A viable substitute for reestablishing environmental health is bioremediation (19).

Microbial treatment for Pb bioremediation has been marketed due to its low cost, environmental friendliness, and high reliability (20). Heavy metals like Pb can be bioremediated by a number of bacterial species. Previously, several multi-metal-resistant bacterial strains such as *Brucella intermedia* (21), *Comamonas testosteroni, Bacillus cereus* (22), and *Acinetobacter haemolyticus* (23) have been isolated and characterized from industrial effluents. Bacteria may metabolize and transform heavy metals into less hazardous forms through a variety of mechanisms, such as intracellular sequestration, metallothioneins, glutathione, and efflux transport (24). Mechanisms such as biosorption, precipitation, and bioaccumulation are employed in bacterial bioremediation (25). Recently, research on *Acinetobacter* sp*.* (HAR20), a Pb-resistant strain, was found in the harbor seawater of Oran and has shown promise in breaking down spent engine oil and eliminating heavy metals (26). This bacterial strain was able to remediate a variety of organic and inorganic pollutants, including pesticides, heavy metals, hydrocarbons, and pharmaceutical compounds, by producing emulsifiers, biosurfactants, enzymes, and phytohormones (27, 28). The ability of *Acinetobacter* species to thrive in polluted environments further reinforces their potential for large-scale environmental cleanup (29). In South Asia, notably Pakistan, *Labeo rohita* is critical to aquaculture and fisheries (30) and acts as a significant bioindicator owing to its ability to accumulate heavy metals in its organs, causing considerable physiological and histological alterations (31). Understanding Pb accumulation in bioindicator species is crucial for assessing pollution levels and the effectiveness of bioremediation strategies (32).

Utilizing *L. rohita* as a bioindicator, the current study aims to determine the efficiency of *Acinetobacter* sp. in reducing Pb accumulation in *L. rohita*. The primary objective of the present study was to isolate and characterize Pb-resistant bacteria from industrial effluent collected from Chokera, Faisalabad. Antioxidant enzyme profiling of isolated bacteria subjected to Pb stress was studied along with metal interaction on the surface of bacterial cells, which was determined using Fourier transform infrared spectroscopy (FTIR). Additionally, intracellular Pb accumulation was confirmed using scanning electron microscopy and energy dispersive X-ray (EDX) analysis. This study also intends to analyze growth, hematobiochemical parameters, enzymatic activities, and histopathological alterations upon Pb accumulation in *L. rohita* and the role of *Acinetobacter* sp. in alleviating these Pb-induced deteriorations.

## MATERIALS AND METHODS

### Sample collection and screening of Pb-tolerant bacterial strain

Thirty-five industrial effluent samples were collected from various locations of Chokera in Faisalabad, Pakistan. Wastewater samples were collected in PVC bottles and stored at 4°C until analysis. Some physicochemical parameters of effluent samples (temperature, pH, and color) were observed at the time of sample collection. Wastewater samples were serially diluted in sterile distilled water from 10^−1^ to 10^−3^ before plating onto Luria-Bertani (LB) agar supplemented with 2 mM Pb(NO_3_)_2_ and incubated for 24–72 h at 37°C. Colonies with discrete morphological characteristics were selected for further study.

### Evaluation of minimum inhibitory concentrations

To determine the minimum inhibitory concentration (33), minimal salt medium broth composed of 1 g (NH_4_)_2_SO_4_, 0.05 g glucose, 0.1 g KH_2_PO_4_, 0.02 g FeSO_4_, 0.1 g MgSO_4_, 0.086 g CaCl_2_, and 0.15 g K_2_HPO_4_ was amended with different concentrations (0.5–35 mM) of Pb (NO_3_)_2_, AsCl_3_, CuSO_4_·5H_2_O, NiCl_2_·6H_2_O, and ZnSO_4_·7H_2_O. The MSM broth was inoculated with a 24-h-old bacterial culture and incubated at 37°C in a rotary shaker at 150 rpm. Each isolate exposed to a specific metal concentration was accompanied by three replicates. However, a blank was taken for every isolate. The MIC is defined as the lowest dosage that fully halts the visible growth of bacteria. The growth of bacterial isolates was assessed by measuring optical density at 600 nm.

### Determination of optimum growth conditions

The parameters for optimal cultivation have been determined. To ascertain the best growth temperature, the bacterial strain PbS1A2 was cultured in a 300 mL flask with 100 mL of LB broth. The bacterial cells were subjected to various temperatures, such as 20°C, 25°C, 30°C, 37°C, 40°C, 45°C, and 50°C, over a period of 24 h. Similarly, to determine the optimal pH, Luria-Bertani broth was adjusted to different pH values (5, 6, 7, 8, 9, and 10), inoculated with the bacterial isolate during its logarithmic growth phase, and incubated in a shaking incubator at 37°C for 24 h, and cell densities were measured at 600 nm.

The growth characteristics of bacterial isolate PbS1A2 were examined both in the presence and absence of Pb. The bacterial strains were grown in MSM broth without the presence of metal serving as a control, as well as in MSM broth medium supplemented with 2 mM Pb for the treated samples. Cell density was calculated at an optical density of 600 nm at regular intervals throughout the 24 h of incubation.

### Biochemical and molecular characterization

The isolated strain PbS1A2, which has been purified, was characterized through standard biochemical tests, including catalase, oxidase, and indole tests, and morphological parameters, including shape, size, margin, motility, and elevation, according to the protocol designed by Cappuccino and Sherman (34). The molecular characterization associated with gene sequencing of the 16S rRNA gene was carried out at Macrogen (South Korea). The BLAST N program was used to conduct a comparison of this sequence to other recognized sequences. The top 10 sequences were selected according to their identity scores. The sequences were aligned using ClustalW, a software program designed for multiple alignments. The strains displaying the highest similarity were selected, and a phylogenetic tree was constructed by employing the MEGA X program.

### Quantification of antioxidant enzymes

To evaluate the activity of antioxidant enzymes, PbS1A2 was cultured in 100 mL of MSM medium at a temperature of 37°C for a duration of 24 h. During this phase of growth, MSM medium was supplemented with 2 mM Pb and subjected to incubation. After 48 h of incubation, the culture was harvested and centrifuged at 14,000 rpm for 10 min, and the pellet was weighed and resuspended in phosphate buffer and sonicated. The supernatant obtained after centrifugation of sonicated pellets was used for the estimation of antioxidant enzymes.

The activity of ascorbate peroxidase (APOX) was assayed according to reference 35. Glutathione transferase (GST) activity was determined following the protocol designed by Habig et al. (33). The activities of peroxidase (POX), superoxide dismutase (SOD), and catalase were assessed by the methods of Reuveni et al., Ewing and Janero, and Beers, Jr., and Sizer (36–38), respectively.

### FTIR, SEM, and EDX analysis

For FTIR analysis, bacterial samples were prepared in compliance with reference 39. In short, bacteria were grown at 37°C for 24 h with and without 2 mM Pb. After a 10-min centrifugation at 3,000 rpm, the bacterial cells were subsequently washed with a saline buffer. Bacterial pellets were then instantly freeze-dried, and their infrared spectra in the 4,000–500/cm range were recorded using an FTIR spectrophotometer. Following the guidelines in reference 40, 2 mM Pb and without Pb bacterial cultures were prepared, and suspensions were dipped on an aluminum stub and numbered. Samples were coated using a sputter coater with a gold film and observed with an energy-dispersive X-ray microanalysis equipment and scanning electron microscopy (IT100LA).

### Preparation of Pb-resistant strain

A Pb-resistant *Acinetobacter* sp. strain was cultured in nutrient broth at 37°C for 24 h to reach a concentration of 1.5 × 10^8^ CFU/mL, following the McFarland standard (41). Bacterial pellets were collected by centrifugation (4,000 × *g* for 10 min) and stored at 4°C for weekly experiments (42).

### Acclimatization of experimental fish

Hundred clinically healthy fish (*L. rohita*), weighing 15–20 g and measuring 7–12 cm in length, were obtained from a local fish farm. Fish were acclimatized for 1 week in well-aerated aquaria (30 cm × 45 cm × 60 cm, 50 L capacity) with continuously monitored water quality parameters (43). They were fed a commercial diet (35% CP) at 4% body weight daily.

### Experimental design

A completely randomized design was employed, dividing 72 fish into three treatment groups. To ensure statistical independence, each treatment group was established in triplicate (three independent aquaria per treatment, with eight fish per aquarium). Water was exchanged (20% daily, 100% weekly), with Pb and bacterial dosages replenished accordingly (Table 1).

**TABLE 1:** Different treatment groups in the experiment, each with triplicate

Group	Treatment
T_0_ (control) + (triplicate)	Fish maintained in Pb-free water
T_1_ (Pb-exposed) + (triplicate)	Fish exposed to 1 mg/L Pb (sublethal dose)
T_2_ (Pb + *Acinetobacter* sp.) + (triplicate)	Fish exposed to Pb and treated with *Acinetobacter* sp*.* (1.5 × 10^8^ CFU/mL)

Feeding was conducted twice daily using a basal diet containing 35% crude protein, ensuring nutritional adequacy at 4% of their body weight as given in Table 2 (44).

**TABLE 2:** Fish feed formulation^*a*^

Ingredient	Percentage
Fish meal	28
Rice polish	16
Canola meal	19
Wheat bran	18
Rice broken	12
Fish oil	5
Vitamin premix	1

^*a*^ See reference 45.

Water quality parameters, including temperature, were measured at 24°C ± 2°C using a mercury-in-glass thermometer. On day one, the pH was roughly 6–7.5, and the dissolved oxygen (DO) concentration was 5–6 mg/L (46).

The physicochemical properties of the water were measured weekly, and DO levels were maintained by air pumps attached to capillaries. Water exchange and tank siphoning were performed daily, and fish health and survival were examined (43).

### Growth performance and sample collection

During the 4-week trial, fish weight and length were measured fortnightly. Wt and *W*_0_ represent the final (on the 28th day) and initial weights (1st day), respectively, and *t* represents the duration of feeding (in days). Percent weight gain (PWG; grams per fish), specific growth rate (SGR), condition factor K, and fish survival rates were estimated (47) using the formulas below


$$SGR=\frac{In\left(Wt\right)-In\left(Wo\right)}{t}\times 100$$

$$PWG=\frac{Wt-Wo}{Wo}\times 100$$

$$K=\frac{Weight\;gain}{Length^{3}}\times 100$$

$$Survival\;rate=\frac{Number\;of\;fish\;at\;the\;end\;of\;trial}{Number\;of\;fish\;at\;the\;start\;of\;trial\;}\times 100$$

### Hematological and biochemical analysis

On day 30, four fish per treatment were anesthetized with clove oil (0.5 mL/L) and sacrificed. Blood samples were collected from the caudal vein using heparinized syringes and divided for hematological (WBC, RBC, Hb, and HCT) and biochemical (ALT, AST, ALP, cholesterol, urea, and creatinine) analysis using a hematology analyzer and MicroLab 300 biochemistry analyzer (48).

### Histopathological examination

Three fish per treatment (each per replicate) were euthanized and dissected. The gills, spleen, liver, heart, and stomach were taken, washed, and stored in formalin. A section of formalin-fixed liver tissue was inserted in designated histology cassettes. The tissues were gradually dehydrated with increasing ethanol concentrations and then embedded in wax. Tissues were removed from wax and sectioned (about 5 µm thick) on a microtome (Histo-line MR 2258) before being mounted on albumenized glass slides. Hematoxylin and eosin staining was used (49, 50). Histopathological examination was performed using a Nikon DS-Fi2 optical microscope, and representative photomicrographs were captured using an attached digital camera. Tissue evaluation was based on qualitative observations of cellular architecture and tissue integrity relative to the control group. No semi-quantitative lesion scoring system or blinded evaluation was employed; therefore, the analysis describes the observed morphological alterations and relative improvements rather than providing statistical quantification.

### Enzymatic activity analysis

#### Sample preparation for enzyme analysis

Proteolytic and amylase activity were assessed in fish livers and digestive tracts. Tissues were homogenized in distilled water (250 mg/mL) at 4°C before centrifugation at 30,000 × *g* for 30 min at 4°C. The resultant supernatant was saved for further enzymatic investigation (51).

#### Protease activity assay

Proteolytic activity was measured using the casein hydrolysis method originally described by Kunitz (52) and later modified by Walter (53) over a pH range of 1.5–10.0 using specific buffer systems. Reaction mixtures containing casein, buffer, and enzyme extract were incubated at 37°C for 1 h. Proteolysis was halted with 8% trichloroacetic acid, followed by chilling at 2°C and centrifugation at 1,800 × *g* for 10 min. The absorbance of the supernatant was measured at 280 nm. Tyrosine was used as the standard, with one unit of activity defined as the enzyme amount releasing 1 µg of tyrosine per minute. This non-specific method was employed previously (54, 55). Total proteolytic activity was calculated by summing the enzyme activities obtained at all pH levels (56).

#### Amylase activity assay

Amylase activity was evaluated by starch hydrolysis using the Somogyi-Nelson colorimetric procedure. Each reaction mixture included 0.125 mL of 2% (wt/vol) starch solution, 0.125 mL of 0.1 M phosphate-citrate buffer (pH 7.5), and 0.05 mL of enzyme extract. Incubations lasted 1 h at 37°C. After incubation, the Somogyi-Nelson technique was used to assess the absorbance at 600 nm. All samples were examined in duplicate (57).


$$\text{Amylase activity (U/mL)}=\frac{\Delta A_{\text{enzyme}}-\Delta A_{\text{blank}}}{\text{incubation time}\times (\text{dilution factor})}\times \text{standard factor}$$

Standard factor = 6.5.

### Pb accumulation analysis

Three fish from each treatment group (T_0_, T_1_, and T_2_) were euthanized and examined. Liver, muscle, and gill tissues were removed, washed, and kept at −20°C. Sample preparation followed reference 58 and FAO Technical Paper No. 212. After partially thawing the frozen samples, 1 g of composite tissue samples was digested at 100°C with 10 mL of concentrated HNO_3_ and 5 mL of H_2_O_2_ until clear. After cooling, the solution was diluted to 50 mL with deionized distilled water. The Pb concentration was determined using an AAS (GPC A932 version 1.1), and the findings were reported as G/g wet weight (59).

### Statistical analysis

Data were analyzed using one-way ANOVA followed by Tukey’s *post hoc* test in SPSS v27. Results are presented as mean ± SD, and graphs were generated using GraphPad Prism 8.0.2.

## RESULTS

### Sampling and screening of Pb-tolerant bacteria

Some physicochemical parameters of industrial effluent were determined at the time of sampling. It was observed that temperature varied from 22°C to 45°C, along with pH ranging from 6 to 9. A total of 40 purified isolated colonies exhibiting differences in margin, shape, elevation, along with apparent characteristics, were screened. Among the 40 purified isolates, only 15 isolates were found to have potential in the medium subjected to 2 mM Pb stress. It was found that the majority of the isolates could resist up to 12 mM. However, the isolate PbS1A2 displayed the highest MIC value, up to 30 mM. Based on Pb tolerance, PbS1A2 was chosen for further research.

### Cross-metal resistance

According to the calculated minimum inhibitory concentration (33) for multiple heavy metal ions, strain PbS1A2 could survive in a wide range of ion concentrations. The PbS1A2 strain revealed that it could resist up to 30 mM Pb and is also capable of resisting other heavy metal ions, including 3 mM (Ni^2+^), 10 mM (Cd^2+^), 5 mM (Zn^2+^), 6 mM (Cu^2+^), and 8 mM (As^3+^). The order of resistance was Pb > Cd > As > Cu > Zn > Ni.

### Biochemical and molecular characterization of bacterial isolate

**Fig 1:**
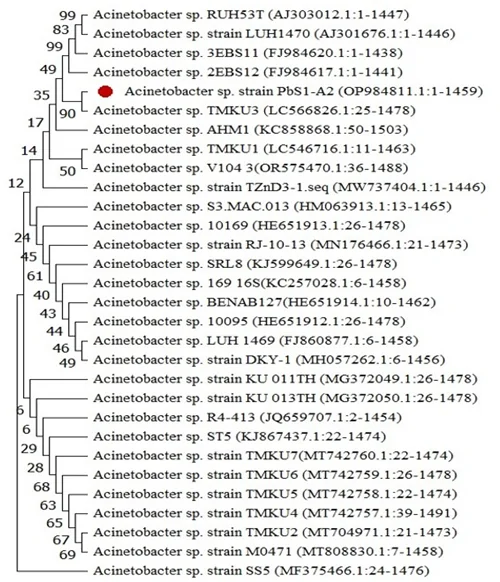
Phylogenetic tree of *Acinetobacter* sp. PbS1A2. The evolutionary relationship of *Acinetobacter* sp. was established with homologous strains. The neighbor-joining method with 1,000 bootstrap test values in MEGA11 software was used to construct the evolutionary history.

Primarily, morphological and biochemical attributes were used to identify the bacterial isolate (Table 3). Based on its identification (100%) as confirmed by BLASTN analysis, the strain PbS1A2 is classified as a member of the genus *Acinetobacter* sp. Using the MEGA11 program, a dendrogram was generated based on similarities among various *Acinetobacter* sp. strains using a bootstrap value of 1,000 (Fig. 1).

**TABLE 3:** Morphological and biochemical characteristics of *Acinetobacter* sp. PbS1A2

Characteristic	Result
Morphological observation	
Colony color	White, shiny
Gram nature	Negative
Colony shape	Round
Edges	Smooth
Elevation	Elevated
Texture	Mucoid
Motility	Non-motile
Biochemical test	
Catalase	Positive
Oxidase	Negative
DNase	Negative
Citrate	Positive
Indole	Negative
Urease	Negative

### Growth conditions of *Acinetobacter* sp. PbS1A2

**Fig 2:**
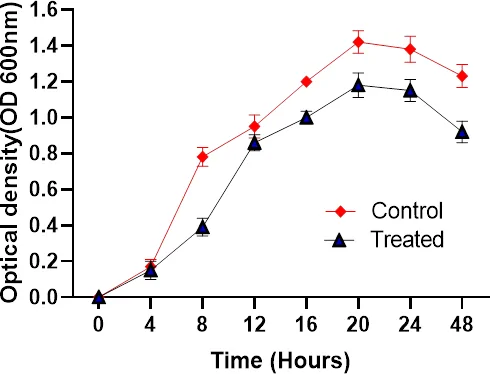
Growth curves of *Acinetobacter* sp. in MSM without Pb (control) and supplemented with 2 mM Pb (treated).

The optimum temperature and pH for *Acinetobacter* sp. were found to be 35°C–45°C and 5.0–10.0, respectively. The growth of *Acinetobacter* sp. was substantially decreased after 2 mM Pb stress compared to the control culture (Fig. 2).

### Quantification of antioxidant enzymes

**Fig 3:**
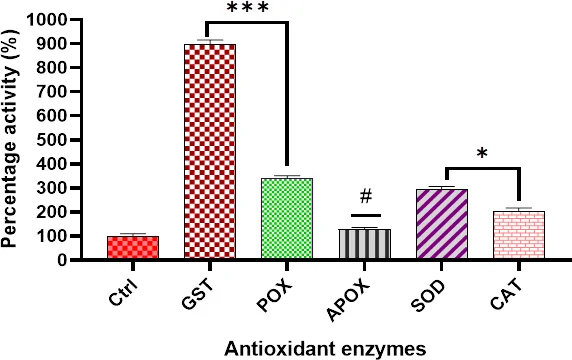
Activity profile of antioxidant enzymes exhibited by *Acinetobacter* PbS1A2 upon exposure to 2 mM Pb, where *P < 0.005, ***P < 0.0001, and #*P* > 0.05.

The evaluation of antioxidant enzymes under Pb exposure provides particularly intriguing results. Pb presence provokes activities of all the antioxidant enzymes. A pronounced rise was observed in GST (795%), POX (229%), SOD (195%), CAT (104%), and APOX (28%) activities (Fig. 3).

### Fourier transform infrared, scanning electron microscopy, and energy-dispersive X-ray analysis

**Fig 4:**
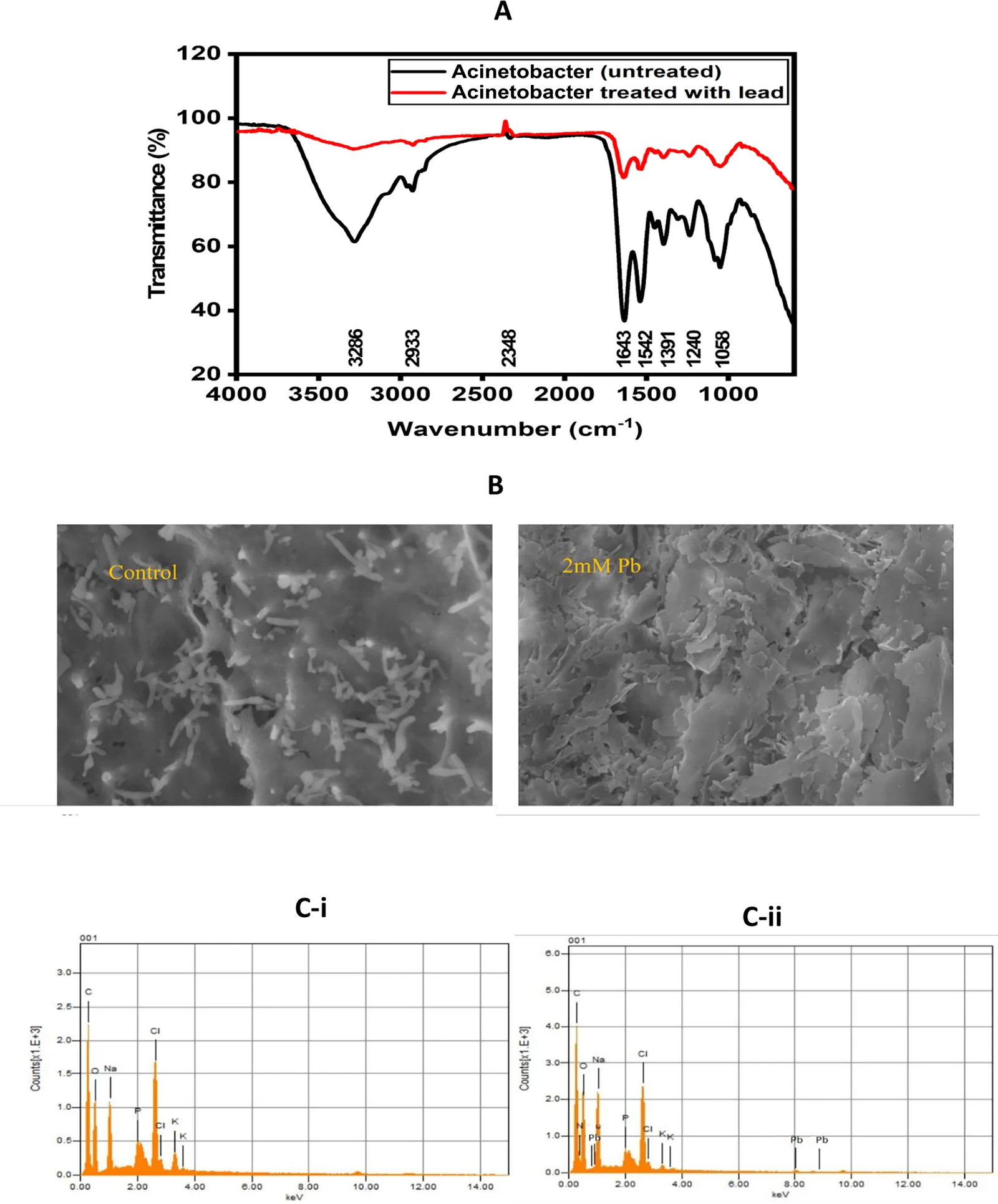
(**A**) FTIR analysis of *Acinetobacter* sp. and images of scanning electron microscopy (**B**) with energy dispersive X-ray of *Acinetobacter* PbS1A2 in the absence (**C-i**) and presence of 2 mM Pb (**C-ii**).

FTIR, SEM, and EDS/EDX spectrum of control and treated bacterial strain *Acinetobacter* sp. PbS1A2 are shown in Fig. 4. The FTIR analysis showed that the peaks of *Acinetobacter* sp. PbS1A2 in metal-treated culture shifted from 1,540–1,529 and 1,055–1,060 (Fig. 4A). SEM examination revealed the alterations in cell shape after exposure to 2 mM Pb (Fig. 4B). The EDS/EDX spectrum of bacteria without Pb stress indicated specific absorption peaks of amino, carboxyl, hydroxyl, and phosphate groups, confirming the existence of these groups on the bacterial cell surfaces (Fig. 4C-i). Bacterial cells exposed to Pb stress showed changes in peaks; the accumulation of Pb in the cells of *Acinetobacter* sp. PbS1A2 has been identified by EDX (Fig. 4C-ii).

Both energy-dispersive X-ray examination and SEM analysis suggest that Pb is absorbed intracellularly.

### Growth performance

Exposure to Pb appeared to be the main cause for the decline in *L. rohita* growth performance. The lowest values for WG (0.464 ± 0.144 g), SGR (0.009% ± 0.0002%), and final weight (14.96 ± 0.10 g) were recorded in the T_1_ group, which received the Pb dose. The group with the highest final weight (16.21 ± 1.24 g), maximum weight gain (1.380 ± 0.362 g), and SGR (0.049% ± 0.0014%) was the control group (T_0_). The growth indicators improved drastically when Pb-exposed *L. rohita* was remediated with *Acinetobacter* sp. (T_2_) compared with T_1_, with the final weight being closer to the control (15.80 ± 0.78 g). The best condition factor was observed in T_0_ (1.025 ± 0.012) and T_2_ (1.017 ± 0.009), which implies that the deviation from the normal was not as significant as it was with T_1_ (1.086 ± 0.015). These data confirm the effective bioremediation of the toxicological effects of Pb on fish growth with *Acinetobacter* sp*.* (Table 4).

**TABLE 4:** Effect of Pb bioremediated strain *Acinetobacter* sp*.* on growth performance of *L. rohita* during 4-week trial^*a*^

Parameter	Value for treatment group:		
	Control (T_0_)	Pb (T_1_)	Pb + *Acinetobacter* sp. (T_2_)
Initial weight (g)	15.53 ± 1.10a	14.75 ± 0.35cd	14.87 ± 0.29cd
Final weight (g)	16.21 ± 1.24b	14.96 ± 0.10cd	15.80 ± 0.78ab
Weight gain (WG)	1.380 ± 0.362a	0.465 ± 0.144b	0.880 ± 0.482d
SGR (%)	0.049 ± 0.0014a	0.019 ± 0.0007b	0.043 ± 0.004a
Condition factor	1.025 ± 0.012a	1.086 ± 0.015b	1.017 ± 0.009a
Survival rate (%)	95	85	98

^*a*^ SGR refers to specific growth rate. Different lowercase letters in the same column show significant differences (*P* < 0.05). Values are presented as mean *±* SD.

### Body composition

Significant variations were observed across several body composition parameters of *L. rohita* across the three treatment groups (T_0_, T_1_, and T_2_). The values of fish body components such as moisture (7.500 ± 0.707d), crude fat (7.500 ± 0.707b), and gross energy (439 ± 3.563b) were lowest in T_1_ (Pb-exposed group) compared to T_0_ (control) and T_2_ (Pb + *Acinetobacter* sp.). While a significant (*P* ≤ 0.05) recovery of these parameters (moisture, 8.500 ± 0.707a,b; crude fat, 11.500 ± 0.707a; and gross energy, 460 ± 0.629c) from Pb-induced deterioration was observed in T_2_. Crude protein content followed a similar trend, with T_2_ (14.92 ± 0.113a) showing higher values than T_1_ (13.96 ± 0.134b). In contrast, dry matter and nitrogen-free extract were higher in the Pb-exposed group (T_1_) compared to T_0_ and T_2_. As expected, the control group (T_0_) generally exhibited the highest values for most components, reflecting a normal physiological range. These results highlight the bioremediation potential of *Acinetobacter* sp*.* in mitigating Pb toxicity and restoring nutritional quality in *L. rohita* (Table 5).

**TABLE 5:** Effect of Pb bioremediated strain *Acinetobacter* sp. on chemical body composition (means ± standard deviations) of *L. rohita^a^*

Parameter	Value for treatment group:		
	T_0_	T_1_	T_2_
Moisture (%)	9.500 ± 0.707b	7.500 ± 0.707d	8.500 ± 0.707ab
Ash (%)	85.0 ± 1.41a	84 ± 1.41a	84.5 ± 0.707a
Dry matter (%)	90.40 ± 0.400b	92.80 ± 0.200a	91.50 ± 0.707ab
Crude protein (%)	14.84 ± 0.226a	13.96 ± 0.134b	14.92 ± 0.113a
Crude fat (%)	11.000 ± 1.414a	7.500 ± 0.707b	11.500 ± 0.707a
Nitrogen-free extract (%)	64 ± 1.895a	70 ± 0.445c	65 ± 1.301a
Gross energy (Kcal g^−1^)	453 ± 4.242a	439 ± 3.563b	460 ± 0.629c

^*a*^ Different lowercase letters indicate statistically significant differences among interaction means, whereas differences among means with the same lowercase letter are statistically non-significant (*P* > 0.05).

### Digestive enzyme assay

Amylase and protease activity in *L. rohita* markedly decreased by Pb exposure in T_1_. Gut amylase decreased from 0.356 ± 0.011a U/mg in control (T_0_) to 0.223 ± 0.007b U/mg in Pb-exposed group (T_1_). Similarly, liver amylase decreased from 464 ± 0.015a in control to 303 ± 0.018b U/mg in T_1_. Protease activity in the liver (77.94 ± 1.49a) and gut (66.065 ± 1.25a) in control decreased to 67.81 ± 1.25b in the liver and 60.02 ± 1.44b in the gut of the Pb-exposed group (T_1_). *Acinetobacter* sp*.* treatment (T_2_) partially restored enzyme activity, with gut amylase and protease increasing to 0.312 ± 0.004c and 64.77 ± 2.22a U/mg, respectively, and liver amylase and protease increasing to 0.414 ± 0.002c and 74.24 ± 1.40a,c U/mg, respectively. These results highlight the bioremediation potential of *Acinetobacter* sp. in mitigating Pb-induced suppression of digestive enzyme activities in *L. rohita* (Table 6).

**TABLE 6:** Influence of Pb bioremediated *Acinetobacter* sp*.* on amylase and protease activities of the liver and gut of *L. rohita* exposed to Pb^*a*^

Group	Gut		Liver	
	Amylase activity (U/mg protein)	Proteolytic activity (U/mg protein)	Amylase activity (U/mg protein)	Proteolytic activity (U/mg protein)
Control (T_0_)	0.356 ± 0.011a	66.065 ± 1.25a	0.464 ± 0.015a	77.94 ± 1.49a
Pb (T_1_)	0.223 ± 0.007b	60.02 ± 1.44b	0.303 ± 0.018b	67.81 ± 1.25b
Pb + *Acinetobacter* sp. (T_2_)	0.312 ± 0.004c	64.77 ± 2.22a	0.414 ± 0.002c	74.24 ± 1.40ac

^*a*^ Different lowercase letters indicate statistically significant differences among interaction means, whereas differences among means with the same lowercase letter are statistically non-significant (*P* > 0.05).

### Hematological parameters

Pb exposure (T_1_) significantly impaired the hematological parameters of *L. rohita*, with reductions in Hb (10.1 ± 0.14 g/dL), RBCs (7.75 ± 0.07 × 10^6^/µL), WBCs (4.65 ± 0.14 × 10^3^/µL), and HCT (32.5% ± 2.12%) compared to the control group (T_0_). Similarly, MCV (41.94 ± 0.49 fL), monocytes (3.7 ± 0.35 × 10^3^/µL), and eosinophils (2.5 ± 0.71 × 10^3^/µL) were significantly lower in T_1_. However, bioremediation with *Acinetobacter* sp*.* (T_2_) resulted in marked recovery, with Hb (12 ± 0.21 g/dL), RBCs (8.5 ± 0.14 × 10^6^/µL), WBCs (8.35 ± 0.21 × 10^3^/µL), and HCT (39.5% ± 0.70%) approaching control levels. Improvements were also observed in MCV (46.47 ± 1.41 fL), monocytes (5.85 ± 0.21 × 10^3^/µL), and eosinophils (3.5 ± 0.70 × 10^3^/µL). Conversely, lymphocyte levels were reduced in T_1_ (18.85 ± 0.49 × 10^3^/µL) and showed minimal recovery in T_2_ (19.15 ± 0.21 × 10^3^/µL). While MCH and MCHC slightly altered across groups, T_2_ demonstrated improved trends. These results highlight the detrimental effects of Pb on hematological health and the partial restoration achieved through *Acinetobacter* sp.-mediated bioremediation (Table 7).

**TABLE 7:** Effect of Pb bioremediated *Acinetobacter* sp*.* on the hematological parameters of *L. rohita*

Parameter^*a*^	Value^*b*^ for treatment group:		
	Control (T_0_)	Pb (T_1_)	Pb + *Acinetobacter* sp. (T_2_)
Hb (g dL^−1^)	12.4 ± 0.4242	10.1 ± 0.1414*	12 ± 0.21
RBCs (×10^6^ µL)	9.25 ± 0.3535	7.75 ± 0.0707*	8.5 ± 0.14
WBCs (×10^3^ µL)	6.6 ± 0.1414	4.65 ± 0.141*	8.35 ± 0.21
HCT (%)	46 ± 1.4142	32.5 ± 2.1213	39.5 ± 0.70*
MCV (fL) (60)	49.73 ± 0.636	41.94 ± 0.4949*	46.47 ± 1.414
MCH (pg)	13.41 ± 2.8991	13.03 ± 2.82	14.12 ± 1.48**
MCHC (g/dL)	26.96 ± 0.5656	31.08 ± 0.3535	30.38 ± 0.14
NEUT (%)	71 ± 0.707	67.5 ± 0.707	75 ± 1.41
LYM (%)	25.3 ± 1.20	18.85 ± 0.49	19.15 ± 0.21
Monocytes (×10^3^/µL)	6.5 ± 0.707	3.7 ± 0.35*	5.85 ± 0.21
Eosinophils (×10^3^/µL)	5.5 ± 0.707	2.5 ± 0.707*	3.50 ± 0.70

^*a*^ RBC, red blood cell; WBC, white blood cell; Hct, hematocrit; Hb, hemoglobin; MCV, mean corpuscular volume; MCH, mean corpuscular hemoglobin; MCHC, mean corpuscular hemoglobin concentration; NEUT, neutrophils; LYM, lymphocytes.

^*b*^ Data are presented as mean ± SD. *P < 0.01, **P < 0.001.

### Blood biochemical parameters

The blood biochemical parameters in *L. rohita* showed significant alteration upon Pb exposure (T_1_). The levels of almost all the parameters of liver function test (LFT), such as AST, ALP, and ALT, were found to be elevated in T_1_ and showed significant variations from the other two groups (T_0_ and T_2_). RFT of group T_1_ was also significantly higher. These biochemical parameters were significantly restored in T_2_ (Pb *+ Acinetobacter* sp.) with LFT (AST [101 ± 0.70 U/L], ALP [1,340 ± 4.24 U/L], and ALT [160 ± 9.89 U/L]) and RFT (urea [65 ± 0.89 mg/dL] and creatinine [0.90 ± 0.00 mg/dL]) approaching control values. Lipid profile (triglycerides [372 ± 0.71 mg/dL] and cholesterol [163.5 ± 9.19 mg/dL]) also showed significant improvement, though not fully restored to control levels. HDL levels were highest in T2 (41.5 ± 2.12 mg/dL), reflecting enhanced lipid balance. These findings demonstrate the potential of *Acinetobacter* sp*.* in significantly mitigating the detrimental effects of Pb on blood biochemical parameters of *L. rohita* (Table 8).

**TABLE 8:** Effect of Pb bioremediated *Acinetobacter* sp. on the blood biochemical parameters of *L. rohita*

Parameter^*a*^	Value^*b*^ for treatment group:		
	Control (T_0_)	Pb (T_1_)	Pb + *Acinetobacter* sp. (T_2_)
LFT			
AST	109.5 ± 0.707	137.5 ± 2.121*	101 ± 0.70**
ALT	186 ± 8.485	212.5 ± 3.535**	160 ± 9.89
ALP	883 ± 9.899	1,391 ± 15.556**	1,340 ± 4.24
RFT			
Urea	54.5 ± 0.707	94 ± 0.707**	65 ± 0.89
Creatinine	0.85 ± 0.070	1.25 ± 0.070*	0.900 ± 0.00
Lipid profile			
Cholesterol	143.5 ± 0.707	163.5 ± 9.1923*	150 ± 0.70
Triglyceride	129.5 ± 0.707	372 ± 0.707***	154 ± 1.41**
HDL	34 ± 1.41	26 ± 0.707	41.5 ± 2.12

^*a*^ HDL, high density lipoprotein; ALP, alkaline phosphatase; LDL, low density lipoprotein; ALT, aspartate aminotransferase; and ALP, alkaline phosphatase.

^*b*^ Values are presented as mean ± SD. *P < 0.01, **P < 0.001, ***P < 0.0001.

### Pb accumulation in tissues

**Fig 5:**
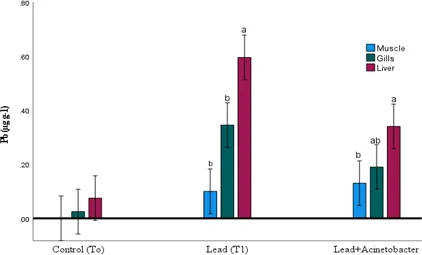
Effect of *Acinetobacter* sp*.* on the Pb levels in tissues of *L. rohita*. Error bars represent the SD from the mean (*n* = 12).

In comparison with the control groups, *L. rohita* in the Pb-only group (T_1_) showed the highest level of Pb in the liver (0.595 ± 0.0212 µg g^−1^), gills (0.355 ± 0.0494 µg g^−1^), and muscles (0.225 ± 0.0353 µg g^−1^). However, treatment with *Acinetobacter* sp*.* (T_2_) reduced Pb accumulation in the liver (0.34 ± 0.028 µg g^−1^), gills (0.19 ± 0.014 µg g^−1^), and muscles (0.13 ± 0.028 µg g^−1^) compared to the T_1_ group. The substantial bioremediation capacity of T_2_ in Pb-contaminated environments was highlighted by the greatly decreased Pb accumulation (*P* < 0.05) in the T_2_ treatment groups compared to T_1_ (Fig. 5).

### Histopathological examinations

**Fig 6:**
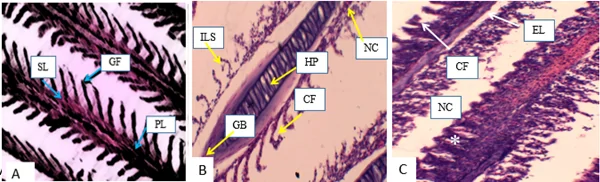
(**A**) The control group (T_0_) showed normal gill structure with primary lamellae (61) and secondary gill lamellae (SL). (**B**) The Pb-exposed group (T_1_) exhibited degeneration, hyperplasia (HP), gill bridging (46), curling filaments (CF), and cellular necrosis (CN). (**C**) The Pb + *Acinetobacter* sp. treatment group (T_2_) showed improved structural integrity, with reduced epithelial lifting (EL) and gill necrosis represented by an asterisk (*).

**Fig 7:**
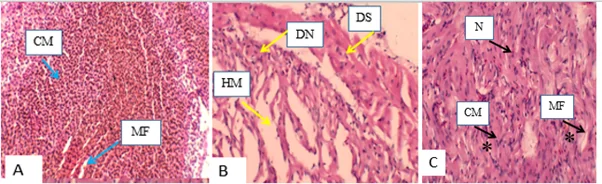
(**A**) T_0_ fish showing a normal nucleus (N)*,* heart myocardial fiber (52)*,* and cardiac muscle (CM). (**B**) T_1_ fish exposed to Pb showed dislocation of the nucleus (DN), edema, infiltration*,* thickening, necrosis, hemorrhage (HM), and dilated sinusoid (DS)*.* (**C**) T_2_ fish showed an appearance of recovery in the normal nucleus (N)*,* heart myocardial fiber (52)*,* and cardiac muscle (CM). Photomicrographs were taken at 40× magnification using a light microscope*.* *, sign of improvement.

**Fig 8:**
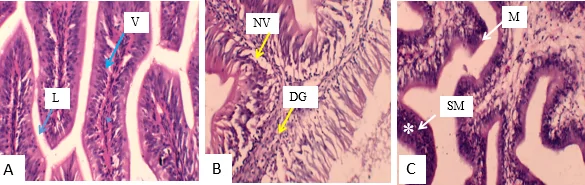
(**A**) Control group (T_0_) displayed normal intestinal villi (V) and lumen (L). (**B**) Pb-exposed group (T_1_) showed necrotic villi (NV) and deformed goblet cells (62). (**C**) *Acinetobacter* sp. treatment (T_2_) showed restoration of mucosal (M) and submucosal (SM) layers, with goblet cells appearing more intact. *, sign of improvement.

**Fig 9:**
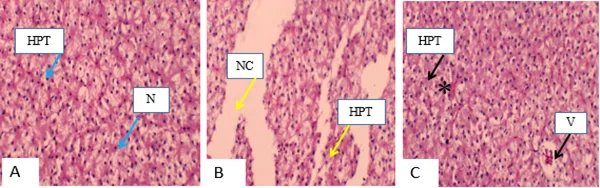
(**A**) Control group (T_0_) displayed normal liver sinusoids, nucleus (N), and RBCs. (**B**) Pb-exposed group (T_1_) exhibited necrosis (NC), hepatocyte vacuolation (HPT), and enlarged cytoplasmic vacuoles (CV). (**C**) *Acinetobacter* sp. treatment group (T_2_) showed partial restoration of liver architecture, with visible improvement in RBC distribution and reduced necrosis. *, sign of improvement.

**Fig 10:**
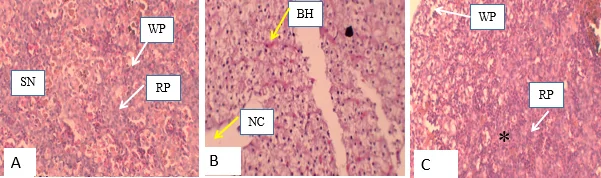
(**A**) Control group (T_0_) showed normal spleen structure with white pulp (WP), red pulp (RP) (63), and sinusoids (SN). (**B**) Pb-exposed group (T_1_) exhibited increased connective tissue, hemorrhage (BH), necrosis (NC), and vacuoles (CL). (**C**) The *Acinetobacter* sp*.* treatment group (T_2_) showed observable improvement, with restored spleen structure and reduced necrosis. *, sign of improvement.

After 30 days of treatment, histopathological changes were observed in *L. rohita* gills, liver, intestine, spleen, and heart. The gills exposed to a sublethal dose (1 mg L^−1^) of Pb in T_1_ revealed degeneration of the central axis, cellular necrosis in gill filaments, and apparent hyperplasia. Gill lamellar curling, aneurysm, and epithelial lifting were also observed. In the T_2_ group (Pb + *Acinetobacter* sp.), the tissues appeared more structurally preserved compared to the maximal damage observed in the Pb group (T_1_). These alterations appeared less pronounced in T_2_ following *Acinetobacter* sp. treatment. These alterations were significantly (*) recovered in T_2_ upon *Acinetobacter* sp*.* treatment (Fig. 6). The T_0_ fish showed normal cardiac tissue with intact nuclei, myocardial fibers, and cardiac muscles. Pb-exposed T_1_ fish showed clear tissue damage, including nuclear dislocation, edema, necrosis, hemorrhage, and dilated sinusoids. In contrast, T_2_ fish showed improved cardiac architecture, indicating recovery from Pb-induced damage (Fig. 7). The intestine in T_1_ (Pb dose of 1 mg L^−1^) showed deterioration of the columnar epithelium, necrosis of villus tips, deformation of basement membranes, and goblet cells. While in T_2_, treatment with *Acinetobacter* sp. was associated with reduced degeneration of the intestinal tissues caused by Pb (Fig. 8). The liver revealed apparent necrosis (NC), hemolysis of hepatocytes (HPT), hepatocyte vacuolation, edema, congestion, and increased sinusoidal space in Pb-exposed fish (T_1_). In T_2_ treatment, the hepatic architecture appeared more comparable to that of the control group, with an observable reduction in necrotic markers. Both bacterial strains had normal cellular architecture (Fig. 9). Cellular infiltration, dilated cytoplasmic vacuoles, moderate necrosis, and tissue degradation were seen in the Pb-only spleen. The spleen tissues in the T_2_ treatment group exhibited evidence of structural restoration compared to the T_1_ group (Fig. 10).

## DISCUSSION

Since industrialization and technology have developed, tremendous quantities of hazardous materials and heavy metals (such as cadmium, chromium, and lead), as well as metalloids and organic pollutants, have been released into the environment, leading to substantial damage to the ecosystem (64). Lead (62) is a non-essential metal that has been proven to be extremely harmful to people, animals, plants, and microbes. The mechanical removal of Pb^2+^ ions is expensive and tedious; using bacteria to accumulate Pb^2+^ ions might be an alternate strategy (65).

This study reports the isolation of a bacterial strain from an industrial effluent sample that is capable of resisting Pb up to 30 mM, and also exhibits tolerance to other heavy metals such as Cd^2+^, As^3+^, Ni^2+^, Cu^2+^, and Zn^2+^. The present study is consistent with the previous study in which Pb-resistant strain of *Acinetobacter* was isolated from the polluted soil to check its bioremediation potential against Pb ions (66). Similarly, another study by Nil et al. (26) reported that *Acinetobacter* sp*.* HAR20 displayed resistance against multiple heavy metals such as Pb, Fe, Cu, Ni, Zn, and Cd up to 600, 300, 200, 150, 100, and 5 mg L^−1^, respectively.

As Pb stress induces reactive oxygen species (1), such as superoxide radicals, hydroxyl radicals, and hydrogen peroxide, which trigger oxidative damage and cell death, it has an immense impact on microbial antioxidant systems (67). While ROS interact with biomolecules, it leads to lipid peroxidation, protein denaturation, and DNA mutation, all of which are toxic to cells (61). The bacteria produce an array of enzymes, notably ascorbate peroxidase, catalase, glutathione transferase, and superoxide dismutase, to combat oxidative stress (68).

Antioxidant enzyme activities in the presence of lead were found in the current study and are displayed in Fig. 3. The antioxidant enzyme profile of *Acinetobacter* sp. PbS1A2 with and without 2 mM Pb stress revealed captivating results. Pb presence provoked activities of all the antioxidant enzymes. A pronounced rise was observed in GST (795%), POX (229%), SOD (195%), CAT (104%), and APOX (28%) activities.

Peroxidases are also considered stress enzymes, as their production can be affected by various environmental stress factors, such as metal ion stress from elements, including Pb, Cd, Zn, and Cu, drought, water, and gamma-radiation stress along with water stress, drought, and gamma radiation (69).

The current results are in association with Elahi et al. (70), who confirmed the significant rise in the production of all antioxidant enzymes under Cr stress. Our results are in good agreement with the findings of Zafar et al. (71), who also noted that Pb stress induces a profound increase in SOD, GST, and APOX. Another study by Bhadekar (72) stated higher production of superoxide dismutase under Pb stress, whereas Zafar et al. (71) demonstrated higher production of SOD and APOX under Cd stress.

The occurrence of functional chains on the active site of the cell wall is essential for biosorption caused by the nonspecific adhesion of Pb+ on the bacterial strain’s cell surface. FTIR analysis of *Acinetobacter* sp. PbS1A2 without Pb stress indicated specific absorption patterns of amino, carboxyl, hydroxyl, and phosphate groups, confirming the existence of the appropriate groups on the bacterial cell surfaces. In contrast, when exposed to Pb stress, visible changes in the peaks at 3,286–2,933 cm^−1^ and 1,700–1,000 cm^−1^ were observed. These findings are consistent with a previous study by Zafar et al. (71), who reported changes in functional groups after exposure to Pb (73).

The infrared peaks found between 3,450 and 3,200 cm^−1^ are distinctive for single bond NH group moieties on the cell surface and hydroxyl groups (74). The distinctive peaks for polysaccharides correlate to the broad bands at 1,100 and 1,000 cm^−1^ (75). Orthophosphate has been identified in the typical band area at 1,100 and 1,030 (PO_4_^3−^). However, C-N stretching can also be seen at the same wavelength in the absorption spectrum, ranging from 1,350 to 1,000 cm^−1^ (76). SEM/EDX analysis was used to assess the Pb accumulation inside *Acinetobacter* sp. cells. During exposure to 2 mM Pb, *Acinetobacter* sp. cells exhibited alterations in shape, which were identified by SEM analysis and verified by EDX. These results are parallel with the findings of Zafar et al. (71), who reported alterations in morphology after exposure to 2 mM Pb.

Pb exposure is a well-documented stressor for fish, causing metabolic stress and impaired respiration, affecting growth through reduced food intake (77). Our findings provide credence to this, demonstrating that fish exposed to Pb (T_1_) exhibited reduced weight, length, SGR, and CF. This is consistent with reference 78, which showed the detrimental effects of Pb (NO_3_)_2_ in *Cyprinus carpio,* resulting in behavioral abnormalities, such as altered feeding and swimming behaviors, that further impede growth. The prominent decrement in CF in T_1_ further indicates a decline in the overall health of Pb-exposed fish. Our research demonstrates the potential of *Acinetobacter* sp*.* supplementation (T_2_) to reduce these harmful effects. When *L. rohita* was treated with *Acinetobacter* sp*.* (T_2_), its growth characteristics were noticeably better than those of the T_1_ group. *Acinetobacter* sp*.* may mitigate heavy metal toxicity in farmed fish as previously proposed by Giri et al. (79) by decreasing metal intake, boosting detoxification, and enhancing the fish’s general physiological state. This beneficial effect is explained by the bacterium’s capacity to improve food absorption and decrease Pb bioavailability (80). This work supports the well-established hypothesis that microbes may eliminate heavy metal contamination from the environment. According to a previous study by Abbasi et al. (78), an isolated strain of *Bacillus* sp. removed over 80% of the Pb from a growth medium. Similarly, *Bacillus coagulans* and *Lactobacillus lactis* were also reported to improve fish growth in metal-exposed environments (44, 79), proposing that a number of probiotics and isolated bacteria have been shown to promote gut health, restore fish growth, lower oxidative stress, and bind and excrete metals to prevent heavy metal toxicity in aquaculture.

Fish exposed to Pb have a lower crude protein, fat, and ash content, which are probably caused by oxidative stress and metabolic disturbance (81). Our results corroborate this by showing a prominent decline in these parameters in Pb-exposed *L. rohita*. Other fish species have also shown comparable detrimental effects of Pb on body composition (82). For instance, Dawood et al. (83) reported that heavy metal exposure in fish led to intestinal damage, such as histological changes, hemorrhage, and vacuolar degeneration, which impaired nutrient absorption, lowering the body composition. Supplementing with *Acinetobacter* sp., however, reversed these adverse effects and improved the body composition of Pb-exposed *L. rohita*. This study aligns with the previous research of Eissa et al. (84), who demonstrated that bacterial bioremediation can significantly lower Pb uptake and restore body composition in fish exposed to heavy metals. The study by Opiyo et al. (85) also demonstrated similar results, showing that inclusion of probiotics like *Bacillus subtilis* significantly increased the protein content, suppressed the crude lipid content, and enhanced the body composition of treated fish. Some *Bacillus* species are also involved in the synthesis of digestive enzymes, antimicrobial compounds, and immunological activation, improving digestion and nutritional absorption in the gut (86). According to the consensus, some bacteria release enzymes like protease that aid in the digestion of proteins and absorption of nutrients (87). Consequently, T_2_ results conclude that *Acinetobacter* sp*.* may also function similarly to these beneficial bacteria, enhancing protein content and general body composition, thereby reducing the harmful effects of Pb.

Substantial decline in the activity of digestive enzymes such as amylase and protease was detected in *L. rohita* upon Pb exposure (T_1_). These enzymes are crucial for the digestion of carbohydrates and proteins. In the past, Kumar et al. (88) have documented a similar decline in enzyme activity in fish under Pb-induced stress. Pb interferes with enzyme synthesis and activity, potentially through oxidative stress and competition for metal ion binding sites (89). This decline observed in our study reflects a wide range of digestive enzyme impairment caused by Pb in *L. rohita*. Our findings are also confirmed by Zafar et al. (90, 91), who reported that heavy metal exposure lowers protease and amylase activities in fish, illustrating the complex effects of heavy metals on digestive processes. The results showed that treating Pb-exposed *L. rohita* with *Acinetobacter* sp. significantly boosted the activity of digestive enzymes. The results of the current investigation are consistent with earlier research by Giri et al. (42), who showed the advantages of bacterial treatments for reestablishing the function of digestive enzymes in fish exposed to heavy metals. Additionally, Wagh et al. (92) proposed that bacteria may be able to increase nutrient absorption by Pb detoxification, thereby enhancing the antioxidant enzymatic activities. In addition to enzyme activity, bacterial treatments have also been shown to improve overall digestive morphology, highlighting their positive influence on fish digestive health (86).

This study opens up new research directions and possible applications in aquaculture and environmental management by proving that *Acinetobacter* sp. is efficient against Pb-induced digestive dysfunction.

In the current investigation, the liver accumulates the highest level of Pb, followed by gills and muscles. This accumulation pattern coincides with the study of Adhikary et al. (93), who demonstrated the liver as a primary heavy metal accumulation site due to its metabolic activity. The liver’s functions of detoxification, metabolism, storage, and redistribution contribute to this accumulation (94). According to recent findings, Pb accumulation in all *L. rohita* tissues significantly decreased in the group that was bioremediated with *Acinetobacter* sp. *Acinetobacter haemolyticus* was another potential bioremediator investigated by Kumar and Singh (23) for the *in vitro* removal of cadmium. Bacteria such as *Aeromonas sobria* (95), *Gemella* sp*.* (96), *Pseudomonas putida,* and *Pseudomonas fluorescens* (97) all demonstrated a notable *in vitro* heavy metal bioremediation potential. However, the utilization of particular bacterial species, such as *Bacillus* and *Arthrobacter*, represents another promising approach for mitigating heavy metal toxicity in fish (98). FTIR, SEM, and EDX analyses confirmed Pb adsorption via phosphate, carboxyl, and amide groups and intracellular Pb accumulation, indicating *Klebsiella pneumoniae* as a promising candidate for industrial effluent bioremediation (15). These bacteria have been shown to enhance health and reduce heavy metal accumulation in fish organs (63). In a similar study, Zhai et al. (99) found that *Lactobacillus plantarum* reduced Pb-induced oxidative stress in *Oreochromis niloticus* by decreasing Pb accumulation in fish tissues. These findings imply that numerous bacterial strains are useful tools for countering heavy metal accumulation in fish.

The hematological parameters of *L. rohita*, such as RBC, WBC, Hb, and HCT, were found to be notably reduced due to Pb. It matches the Pb-induced anemia and immune suppression previously reported by Abbasi et al. (78) in common carp. A comparable reduction in RBC and HCT was noted with elevated levels of chromium poisoning in *L. rohita* as reported by Chaudhary et al. (32). The decrease in hematocrit noted in our research might stem from hemodilution caused by an osmoregulatory imbalance, as indicated by Chaudhary et al. (32). On the other hand, treatment with *Acinetobacter* sp. markedly attenuated these Pb-induced hematological changes in *L. rohita*. In a study, Yin et al. (100) discovered that dietary *B. subtilis* administration efficiently protects the immune system of Pb-exposed fish through the normalization of hematological parameters and restoration of δ-ALAD levels. All these results corroborate with our results and consequently suggest that *Acinetobacter* sp. may be used as a protective agent in the treatment of invasive Pb damage in fish hematology.

Elevated blood biochemical markers (urea, creatinine, AST, ALT, ALP, cholesterol, and LDL) observed in Pb-exposed *L. rohita* in the current study are indicative of metabolic disturbances and organ damage observed in *Potamotrygon magdalenae* (101). Similar alterations were previously observed with chromium exposure on the same species (*L. rohita*) reported by Ray et al. (90). These findings are in line with the findings of Reda et al. (102), who demonstrated that *O. niloticus* exposed to a mixture of Pb, Hg, and pendimethalin showed comparable increases in serum creatinine, urea, and liver enzymes.

These Pb-induced biochemical alterations were dramatically reversed by *Acinetobacter* sp. treatment, returning biochemical levels to normal. This is consistent with research by El-Kady et al. (103), who demonstrated that bacterial treatments improve certain fish parameters exposed to metals. Together, our results demonstrate how bioremediation and other mitigation techniques can abate the harmful impacts of heavy metals on fish biochemical profiles.

Histopathological alterations such as gill filament degeneration, intestinal atrophy, liver necrosis, cardiac muscle congestion, and spleen tissue necrosis were observed in the Pb-exposed group. These histopathological deteriorations align with prior findings of Mahi et al. (82), who reported dose-dependent alterations in tilapia, including gill lamellar, intestinal villus disintegration, and vacuolization. Additionally, Ali et al. (3) reported hypertrophy and epithelial necrosis in the gills, tubular damage, hepatocyte degeneration, and necrosis in the liver. While this study employed qualitative histopathological assessment rather than semi-quantitative scoring, *Acinetobacter* sp. reversed Pb-induced histopathological damage in *L. rohita* by displaying improvements in the morphological structures of the intestine, liver, and gill tissues, as well as decreased Pb accumulation. These improvements are consistent with the findings of Zannat et al. (98), who showed that different probiotic bacterial strains can mitigate these heavy metal-induced histopathological abnormalities. This suggests that *Acinetobacter* sp. can overcome the harmful effects of Pb on fish. Numerous bacteria, including *Lactobacillus reuteri* and *B. coagulans*, have been shown to remediate Pb buildup in fish tissues, further validating our findings (42, 104). These results imply that *Acinetobacter* sp*.* may be an advantageous bioremediation technique to lower Pb bioavailability and enhance fish health in contaminated areas, although the exact mechanisms by which this bacterium functions require further investigation. Future studies utilizing standardized lesion scoring systems will be necessary to provide a quantitative validation of these histological improvements.

### Conclusion

In the current study, the optimum temperature and pH for *Acinetobacter* sp. were found to be 35°C–45°C and 5.0–10.0, respectively. The PbS1A2 strain could resist up to 30 mM Pb and other heavy metals.

This study reports a clear increase in GST (795%), POX (229%), SOD (195%), CAT (104%), and APOX (28%) activities. Additionally, this research also investigates the bioremedial potential of *Acinetobacter* sp. for reducing Pb toxicity in *L. rohita*. Fish subjected to a sublethal concentration of Pb (1 mg/L) in T_1_ demonstrated considerable physiological and health-related issues. These included diminished survival rates, inhibited growth, changes in body composition, decreased activity of digestive enzymes, alterations in hematological and biochemical profiles, as well as histological damage. However, introducing *Acinetobacter* sp. at a concentration of 1.5 × 10^8^ CFU/mL (T_2_) significantly improved these effects. Improved survival rates, increased growth performance, restored body composition and digestive enzyme function, and minimized Pb-induced hematological, biochemical, and histological damage were observed in T_2_.

These findings suggest that *Acinetobacter* sp. reduced Pb bioavailability, thereby facilitating the recovery of *L. rohita* under the experimental conditions of this study. Therefore, *Acinetobacter* sp. may serve as a potential candidate for the bioremediation of Pb-contaminated aquatic systems. However, further studies are required to elucidate its underlying mechanisms and evaluate its biosafety, long-term effects, and field applicability before practical implementation.

## Data Availability

The sequence of the 16S rRNA gene from strain PbS1A2 has been deposted in the NCBI GenBank database under accession. no. OP984811.1.
